# Promoting Health Education through Mobile Apps: A Quantitative Analysis of American Hospitals

**DOI:** 10.3390/healthcare10112231

**Published:** 2022-11-08

**Authors:** Pablo Medina Aguerrebere, Eva Medina, Toni Gonzalez Pacanowski

**Affiliations:** 1 Faculty of Communications, Arts, and Sciences, Canadian University Dubai, Dubai 117781, United Arab Emirates; 2 School of Communication and Psychology, University of Alicante, 03690 Alicante, Spain

**Keywords:** hospitals, corporate communication, health education, mobile apps, patients’ empowerment

## Abstract

Using mobile apps as a corporate communication tool helps hospitals to improve their health education initiatives. This paper aims to analyze how these organizations can use mobile apps to implement health education initiatives addressed to patients. To achieve this, we conducted a literature review (health education, mobile apps, role of doctors and patients), and we resorted to using 38 quantitative indicators to evaluate how the 100 best hospitals in the United States manage mobile apps for implementing health education initiatives addressed to patients. Our results prove that 95% of hospitals displayed general mobile apps for patients, but just some of these organizations proposed mobile apps for patients suffering from non-communicable diseases, including: heart diseases (9.47%), cancer (7.37%), chronic respiratory diseases (3.26%), and diabetes (3.16%). We concluded that hospitals should create a department specializing in designing mobile apps that are adapted to patients’ medical and social needs, and that are also consistent with public health priorities.

## 1. Introduction

Health education has become a strategic area for hospitals interested in establishing better relationships with stakeholders, including: patients, employees, suppliers, media companies, public authorities, etc. [1]. These organizations resort to different tools, such as artificial intelligence, big data, websites, patient portals, social media platforms, and mobile applications, to design integrated education experiences allowing patients to reinforce their empowerment [2]. Among all these tools, mobile apps are especially useful for patients, allow them to upload medical information, check test results, track medical metrics, manage appointments with health professionals, conduct video consultations with doctors, etc. [3]. However, many hospitals do not adapt these mobile apps to their patients’ needs, which constitutes a problem from a public health perspective. This paper aims to better understand how hospitals manage mobile apps to implement health education initiatives addressed to patients. To achieve this, we conducted a literature review on health education, the role of health professionals and patients, health education during crises, and the impact of mobile apps on health education. Then, we resorted to using 38 key performance indicators to analyze how the best 100 hospitals in the United States use their mobile apps for health education initiatives addressed to patients, including those suffering from non-communicable diseases. Finally, we presented our quantitative results, as well as three research avenues and three conclusions.

## 2. From Patients’ Education to Health Education Based on Mobile Apps

### 2.1. A Multidisciplinary Approach to Health Education

Health education initiatives contribute to improving peoples’ well-being, hospitals’ internal functioning, and public health authorities’ reputation [4]. That is why many organizations promote this area, such as hospitals, public authorities, and patient associations, as well as schools, highs schools, and higher education institutions [5]. *Schools* resort to creative initiatives (online training for professors, social-media-based learning initiatives for students, etc.) to promote health education among their internal and external stakeholders [6]. *Higher education institutions*, especially schools of medicine and nursing, implement health education initiatives to reinforce their students’ skills in prevention and scientific dissemination [7]. These organizations prioritize values such as empathy, respect, and mutual understanding to help future doctors and nurses to interact with patients more efficiently [8]. These values are especially important for students in the schools of nursing because, on the one hand, these professionals interact with patients with different social backgrounds [9] and, on the other hand, because they must provide patients with an integrated medical service including psychosocial support, empathy, and emotional intelligence [10].

Schools of medicine and nursing, high schools, elementary schools, public authorities, hospitals, and patients’ associations should work in a coordinated way to promote health education and enhance citizens’ well-being [4]. Additionally, these organizations should collaborate with experts from different professional areas, including: communication, economics, engineering, etc. [5]. Among all these areas, ethics is the most important one. Health organizations must view ethics through the prism of care, but also through that of education, as patients are becoming more and more demanding concerning their self-determination and their capacity to take care of themselves [11]. Thanks to ethics, hospitals can implement a multidisciplinary approach that makes their health education initiatives more interesting for all stakeholders, especially for patients [6]. On the other hand, promoting a multidisciplinary approach in health education involves using different formats and tools. Health organizations can resort to storytelling-based communication strategies to promote patients’ involvement with health education campaigns [12]. They can also implement health education initiatives based on performance and arts to interact with stakeholders in a more creative way [13]; they can combine videos, pictures, and online events to train patients on healthy habits [14]; and finally they can also utilize social media platforms to combat misinformation, exchange social support in online communities, and implement medical interventions [15].

### 2.2. Role of Hospitals, Doctors, Nurses, and Patients

Many organizations promote health education (public authorities, patients’ associations, media companies, etc.); however, hospitals remain the most important ones because they represent scientific credibility [11]. Hospitals carry out four main health education initiatives. *First*, they promote health professionals’ involvement in this area to help patients in a more efficient way [16]. *Second*, they train doctors and patients to efficiently participate in collective decision-making processes concerning the latter’s health [17]. *Third*, they define patients’ empowerment at each level of the care production chain to efficiently implement a new medical approach based on health education [18]. Additionally, *fourth*, they resort to standard measurement tools to make sure patients understand medical information, and they improve their medical outcomes [16,19].

Hospitals’ health education campaigns are mainly based on health professionals’ involvement: the latter respect human values (compassion, empathy, etc.), patients’ expectations, and legal frameworks to efficiently promote health education [20]. Promoting these human values positively contributes to making health education campaigns more efficient [21], especially when these campaigns aim to influence ethnic minorities with different cultural backgrounds [22]. Integrating human values into health education initiatives is essential for not only doctors, but also nurses. These professionals interact with several patients facing different situations from a medical and emotional perspective [23], which means that they must provide patients with psychosocial and medical support [14].

Hospitals, doctors, and nurses resort to health education to help patients to access medical information, change their attitudes, explain their feelings, and modify their behaviors [24]. Thanks to health education, patients develop the skills needed to make decisions and contribute to their care process; in other words, health education helps patients to reinforce their empowerment [16]. This last concept means that patients can share their opinions, experiences, and expectations with other patients, as well as with health professionals, to make informed decisions [25]. Empowered patients decide whether to be treated or not, whether to be hospitalized or not, and whether to be active or not; being empowered does not necessarily imply making decisions on a health topic that is related to the guidelines imposed by health professionals [18]. Thanks to empowerment, patients and their relatives reinforce their rights [26].

### 2.3. Health Education in Crises

Promoting health education constitutes a social priority, especially when people face pandemics, as was recently the case with the COVID-19 outbreak [27]. To efficiently manage this pandemic, three main types of organizations were involved: international organizations, governments, and citizens [28]. International organizations worked together to analyze test results, symptoms, and treatments, and based on that, they implemented health education campaigns to curve the COVID-19 pandemic [29]. Governments implemented an integrated health education approach based on mutual trust, calling for collective responsibility and coordination of all stakeholders to protect people against this disease [30]. Finally, citizens made a great effort to read medical information about COVID-19, and in this way protected themselves and limited the spread of the infection [31]. Additionally, they respected different prevention measures, such as social distancing, isolation, PCR tests, vaccines, and masks [32]. International organizations, governments, and citizens were not the only ones to actively fight COVID-19. Health professionals played the most important role: they worked hard to help patients accept new medical protocols and promote healthy habits [28]. Unfortunately, many of these professionals did not receive enough support from their organizations—in the form of security measures, financial help, or psychological support—which made it difficult for them to efficiently implement health education initiatives [33].

### 2.4. Promoting Health Education through Mobile Apps

Health organizations resort to different initiatives to promote health education, such as corporate events, media relations, conferences, websites, social media platforms, and mobile applications. Some hospitals integrate mobile apps into their different protocols to enhance patients’ adherence to treatment, disseminate disease-related information, and monitor patients’ medical outcomes [34]. When hospitals resort to these apps, they consider three main criteria: patients’ perceptions, effort expectancy, and social influence [35]. Mobile apps allow hospitals to change the way they deliver healthcare knowledge [36], improve their relations with patients, especially those having difficulties reaching healthcare services due to distance or costs [2], and reinforce their scientific credibility by disseminating accurate data that positively influence patients’ medical outcomes [36].

To efficiently promote mobile apps as a health education tool, hospitals need to train doctors, nurses, and patients on how to use these platforms for educational purposes [35]. Thanks to mobile apps, doctors can interact more often with patients, especially with those suffering from chronic diseases [37], and nurses can monitor patients’ medical outcomes more efficiently [38]. On the other hand, patients using mobile apps become more autonomous and participate more actively in collective decision-making processes concerning their health, which positively influences their medical outcomes [35]. In other words, thanks to mobile applications, patients reinforce their empowerment and protect their rights [39].

More and more hospitals are resorting to mobile apps to implement advanced patient education programs that ensure person-centered care [26]. These programs are especially useful for patients suffering from non-communicable diseases such as diabetes, cancer, heart disease, or chronic respiratory disease. Thanks to mobile apps, doctors and nurses can propose a more integrated medical service for patients suffering from *diabetes*, involving: organizing social support groups, monitoring medical outcomes [40], and disseminating evidence-based educational content [2]. Concerning *cancer* patients, they can use mobile apps to interact with oncologists, read about cancer treatments, and better understand preoperative requirements [41]. Facing surgery constitutes a risk for patients, regardless of their disease; that is why hospitals should integrate mobile apps into their medical protocols to improve patients’ experiences and facilitate doctors’ duties [3].

## 3. Methodology

To better understand how American hospitals manage mobile apps for health education purposes, we resorted to the *World’s Best Hospitals 2021*, a reference study published by *Newsweek* and *Statista* that evaluates hospitals’ medical performance. These two institutions analyzed 2.000 hospitals from 25 different countries (United States, United Kingdom, Germany, Canada, etc.) according to three main criteria: (a) recommendations from more than 74.000 medical experts (doctors, managers, healthcare professionals); (b) patient satisfaction surveys (medical care, organizational services, etc.); and (c) medical indicators (quality of treatments, hygiene measures, number of patients per doctor, etc.). Recommendations from medical experts accounted for 55%, patient experience 15%, and medical indicators 30%. Finally, all results obtained were validated by an independent global board of medical experts from different countries, such as the USA, Switzerland, Israel, and Germany. (More information about this methodology is available here: https://d.newsweek.com/en/file/461200/worlds-best-hospitals-2021-extended-methodology-20200303.pdf. Document retrieved on 24 August 2022.).

Thanks to this ranking, we identified the 100 best hospitals in the United States (see *Appendix A. List of best hospitals in the USA*). Afterward, we analyzed how these hospitals managed their corporate websites, patient portals, social media platforms, and mobile apps to promote health education. We focused on corporate websites and patient portals because both platforms allow hospitals to reinforce patients’ engagement with health education initiatives [42]. As for social media platforms, we considered them because they are a useful tool for hospitals’ health education campaigns, allowing for: disseminating information, organizing online consultations, etc. [43]. Finally, we analyzed mobile apps because these tools allow hospitals to make their health education campaigns more dynamic and creative [28]. Additionally, thanks to these apps, patients can control medical metrics and improve their healthcare outcomes [44]. Given the increasing importance of mobile apps in the healthcare industry, we decided to focus our research on these tools.

To analyze how the best US hospitals managed mobile apps for health education purposes, we carried out a quantitative analysis from 4 September to 9 October 2022. We resorted to 38 key performance indicators grouped into four main categories: (a) online integration, (b) general apps for patients, (c) mobile apps for other targets, and (d) mobile apps for patients suffering from non-communicable diseases (see Table 1). We considered non-communicable diseases (NCDs) because they kill 41 million people each year, accounting for 74% of all deaths globally. We analyzed apps for patients suffering from cardiovascular diseases, cancer, chronic respiratory diseases, and diabetes because these four diseases account for over 80% of all premature NCD deaths [45]. To determine the mobile apps used for this purpose, we analyzed every hospital’s corporate website to check if they displayed mobile apps addressed to patients; moreover, we consulted the *Apps Store* and *Google Play* to confirm our results. Once all apps were identified, we analyzed them according to different indicators (see Table 1) that were previously gathered from our literature review. To analyze each app function, we read its online corporate description and we confirmed our results by installing each app on our smartphone. However, many functions were only available for patients admitted in the hospital; that is why we could not analyze all functions, only the most important ones.

We only analyzed hospitals’ corporate profiles (websites, patient portals, social media platforms, mobile apps), and not any other kind of profile (medical departments, events, etc.). Concerning mobile apps, we focused on apps addressed to patients, but not on those used only by health professionals. Additionally, we only evaluated apps developed by the hospital, or apps developed by external providers but adapted to the hospital’s system; in other words, we did not consider external apps recommended by hospitals that were not integrated into the organization’s medical system. Lastly, all indicators were analyzed according to the binary system, except for one that was evaluated as an absolute number: online integration (5. Number of mobile apps).

## 4. Results

Most American hospitals resort to mobile apps to implement health education initiatives. Nevertheless, not all of them manage these platforms in the same way. For example, some organizations belonging to the same hospital group use the same mobile apps —general mobile apps and mobile apps for patients suffering from non-communicable diseases (see *Appendix B. Hospitals using the same mobile apps*). On the other hand, some hospitals use general apps that are developed by external companies but adapted to the hospital’s medical system (see *Appendix C. General apps developed by external companies*). To better understand how the best American hospitals manage mobile apps for health education purposes, we present our quantitative results, grouped into four main categories: (1) online integration, (2) general apps for patients, (3) mobile apps for other targets, and (4) mobile apps for patients suffering from non-communicable diseases.

*1. Online integration*. All hospitals analyzed had a corporate website, a patient portal and a corporate profile on at least one social media platform (*Facebook*, *Twitter*, *YouTube, etc.*). On the other hand, 95% of hospitals displayed at least one mobile app. On average, hospitals proposed 3.72 mobile apps. The best hospitals according to the number of mobile apps were *The Johns Hopkins Hospital* and *Johns Hopkins Bayview Medical Center* (see Table 2 below). These quantitative results prove that 95% of American hospitals integrated websites, patient portals, social media platforms, and mobile apps for health education purposes.

*2. General apps for patients*. According to our results, 95% of hospitals displayed a general app for patients. Hospitals proposed different services thanks to these apps: reviewing test results and medical records (100%), communicating with doctors (100%), uploading personal health data (98.95%), managing appointments with doctors (98.95%), requesting prescriptions (70.53%), paying bills (69.47%), accessing family’s health information (45.26%), finding physicians (35.79%), and conducting video consultations with doctors (32.63%). Most hospitals (72.63%) met between six and seven criteria (see Table 3), and only four of them fulfilled the nine criteria evaluated (see Table 4).

*3. Mobile apps for other targets*. Some hospitals proposed mobile apps for different targets, such as patients facing particular diseases, including depression, obesity, etc. (40%), employees (38.95%), or media companies (4.2%). However, no hospital displayed a mobile app for their suppliers (logistic companies, pharmaceutical organizations, etc.). On the other hand, the only hospitals proposing apps for at least three different targets (media companies, employees, and patients suffering particular diseases) were *Cleveland Clinic*, *Cleveland Clinic Fairview Hospital*, and *Cleveland Clinic Florida*. Finally, 20 hospitals had apps for two different targets; 31 hospitals, for one target; and 46 hospitals did not propose any app for these particular targets.

*4. Mobile apps for patients suffering from non-communicable diseases*. Our results prove that 9.47% of hospitals displaying mobile apps proposed at least one app for patients facing *cardiovascular diseases*. These apps allowed these patients to access health education information (100%), contact doctors (100%), and track medical metrics (22.22%). However, these apps did not allow patients to conduct online consultations with doctors or request prescriptions. The only app meeting at least three out of five criteria was the Corrie Health App (*The Johns Hopkins Hospital*, *Johns Hopkins Bayview Medical Center*). The other apps addressed to patients suffering from cardiovascular diseases only met two criteria: the Mayo Clinic Cardiovascular CME App (*Mayo Clinic*—Rochester, Phoenix, Jacksonville, Health System In Eau Claire), Duke CPR App (*Duke University Hospital*), Vanderbilt Heart and Vascular App (*Vanderbilt University Medical Center*), and TGH Cardiovascular Protocols App (*Tampa General Hospital*).

Concerning *cancer*, only 7.3% of hospitals having mobile apps proposed at least one app for this kind of patient. Thanks to these apps, cancer patients could read medical information (100%), contact doctors (57.14%), and track medical metrics (14.29%); however, these apps did not allow them to conduct online consultations or request prescriptions. The UM Skin Check App (*University of Michigan Hospitals—Michigan Medicine*) was the only app fulfilling three out of five criteria. The other apps addressed to this kind of patients only met two criteria (UCSF Fetal Treatment Center App—*UCSF Medical Center*, UTSW Cancer Clinical Trials App—*UT Southwestern Medical Center*, UPMC Hillman Trials Finder App—*UPMC Presbyterian & Shadyside*) or just one (VeloSano App—*Cleveland Clinic*, *Cleveland Clinic Fairview Hospital*, and *Cleveland Clinic Florida*.)

As for *chronic respiratory diseases*, only 3.16% of hospitals analyzed displayed an app for these patients. These apps allowed them to read medical information (100%), track medical metrics (100%), contact doctors (100%), and carry out online consultations (100%), but they could not order prescriptions (0%). The Sleep App (*Cleveland Clinic*, *Cleveland Clinic Fairview Hospital*, *Cleveland Clinic Florida*) was the only app in this category.

Finally, only 3.16% of hospitals having mobile apps proposed an app for patients suffering from *diabetes*. Thanks to these apps, these patients could read medical information (100%), track medical metrics (100%), and contact doctors (100%). However, no hospital proposed an app allowing them to sign up for online consultations with doctors or request prescriptions. The LDL Cholesterol Calculator App (*The Johns Hopkins Hospital*, *Johns Hopkins Bayview Medical Center*) and Diabetes Emoticons App (*University of Michigan Hospitals—Michigan Medicine*) were the only apps in this category.

## 5. Discussion

Hospital health education initiatives addressed to patients should consider different stakeholders, such as doctors, patients, relatives, etc. [4]; focus on these stakeholders’ needs, including medical outcomes and social support [46]; and integrate different formats, such as storytelling or emotional branding [12]. This integrated approach is essential to allow patients and doctors to engage in medical decision-making processes [47]. Our results prove that most hospitals in America make an effort to implement this integrated approach; in fact, 95% of these organizations displayed, at the same time, a corporate website, a patient portal, different social media platforms, and at least one mobile app. However, out of the 95 hospitals using mobile apps, 31 did not develop their own applications and resorted to external companies; 29 hospitals implemented *My Chart*, an app developed by *Epic*; and 2 hospitals used *Follow My Health*, an app commercialized by *All Scripts*. Additionally, many hospitals, such as *Mayo Clinic Phoenix*, *Inova Alexandria Hospital*, and *Brigham And Women’s Faulkner Hospital*, did not have a mobile app, and used apps implemented by other hospitals belonging to the same hospital group. When hospitals develop their own mobile apps, they can implement efficient health education campaigns based on a more integrated approach (mobile apps, websites, patient portals, and social media platforms).

Besides an integrated approach, hospitals should promote human values. Doctors, nurses, and patients should respect different values, such as compassion, multiculturalism, customization, and ethics, and push hospitals to integrate these values into their online health education initiatives [1]. Respecting human values means that hospitals follow ethical principles [11] and that health professionals consider patients’ expectations and beliefs [20]; moreover, these human values should also be integrated into the hospital’s medical protocols [21]. These human values must also be present within hospitals’ mobile apps. However, our results certify that many hospitals can still improve in this area; on the one hand, 67.35% of hospitals having a general mobile app for patients did not allow them to carry out personal video consultations with doctors and nurses, and on the other hand, 64.21% of hospitals’ mobile apps did not permit patients to find physicians’ contact details (email, phone number, etc.). Hospitals should evolve from a management approach focused on health information (test results, personal health data, appointments) to a more humanistic approach that considers patients’ needs in terms of social and emotional support. This humanistic approach should lead hospitals to develop mobile apps addressed to stakeholders that also play a key role in health education, such as media companies or suppliers. However, our results prove that most hospitals are not following this logic: no hospital displayed an app for suppliers, and only 4.2% of them had an app for media companies.

Integrating mobile apps and human values to reinforce patients’ skills in health education constitutes an opportunity to make hospitals’ brands more credible. These health education initiatives are especially important when hospitals interact with patients facing non-communicable diseases (heart diseases, cancer, diabetes, and chronic respiratory diseases). Patients suffering from heart disease have to establish an open dialogue with doctors based on honesty and non-judgment [48]; patients facing diabetes need to reinforce their skills in health education to improve their medical outcomes [49]; lastly, cancer patients require accurate information about diseases and treatments [50]. Nevertheless, according to our results, most hospitals do not focus on patients facing non-communicable diseases, and only some of them proposed an adapted mobile app for these patients, including those with: cardiovascular diseases (9.47%), cancer (7.37%), and chronic respiratory disease and diabetes (3.16%). When hospitals invest in patients’ needs (knowledge, social help, emotional support), they become more credible, especially when they focus on patients facing serious diseases such as cancer, diabetes, heart disease, or chronic respiratory disease.

Most hospitals analyzed resorted to mobile apps to implement health education initiatives, allowing them to enhance their relationships with stakeholders, especially patients. Despite the interesting ideas explained in this paper, we must highlight three main limitations: (a) a lack of information about how American hospitals integrated mobile apps into their medical protocols, (b) absence of input concerning patients’ perceptions about the use of mobile apps for health education, and (c) difficulty finding other papers focused on this area that would allow us to compare our results. Finally, we recommend researchers interested in this topic focus on three main areas that will be especially relevant in the coming years: (a) integration of mobile apps into hospitals’ internal protocols, (b) impact of big data and artificial intelligence on mobile applications addressed to patients, and (c) design of training sessions to help patients reinforce their empowerment through mobile apps (medical knowledge, technological expertise, and human values).

## 6. Conclusions

Hospitals resort to different technological tools (artificial intelligence, big data, mobile apps) to accelerate their digital transformation, improve their internal functioning, and enhance their patients’ satisfaction with medical treatments. However, these organizations should not only focus on treating patients and leading research: they must also promote health education. Additionally, to achieve this, using technological tools such as mobile apps can become a true advantage. This article aimed to evaluate how hospitals manage their mobile apps for health education purposes. Our quantitative results prove that many of these organizations’ general mobile apps for patients provide several basic services (reviewing test results—100%, managing appointments—98.95%, paying bills—69.47%), but not true health education initiatives, such as conducting video consultations with doctors to allow these them to treat and educate patients at the same time (32.63%). In other words, American hospitals can still improve in this area, and to achieve this, we propose three last ideas. *First*, these organizations should create a department integrating doctors, engineers, experts in corporate communication, and patient representatives to develop mobile apps adapted to patients’ needs and are also consistent with the hospital’s requirements. *Second*, these mobile apps should focus on social and medical content that is useful for patients (medical information about treatments, emotional support groups, etc.) rather than commercial information (hospital’s internal policies, prices of treatments, etc.); in other words, mobile apps must become a tool for reinforcing patients’ empowerment, and not a commercial tool for accelerating hospitals’ business. *Third*, hospitals should collaborate with public authorities and international NGOs (*Ministry of Health*, *World Health Organization*, *International Committee of the Red Cross*, etc.) to develop apps that could also fix some of the most important public health challenges, which includes taking care of patients suffering from non-communicable diseases.

## Figures and Tables

**Table 1:** Key performance indicators.

Online Integration	General Apps for Patients	Mobile Apps for Other Targets	Mobile Apps for Patients Suffering from Non-Communicable Diseases
Corporate website Patient portal Social media platforms Mobile apps Number of mobile apps	Review test results Upload personal data Access family’s health data Communicate with doctors Manage appointments Request prescriptions Video consultations Find physicians Billing	Patients facing particular diseases Employees Suppliers Media companies	Cardiovascular diseases Health education information Track patients’ metrics Contact doctors Online consultations Request prescriptions Cancer Health education information Track patients’ metrics Contact doctors Online consultations Request prescriptions Chronic respiratory diseases Health education information Track patients’ metrics Contact doctors Online consultations Request prescriptions Diabetes Health education information Track patients’ metrics Contact doctors Online consultations Request prescriptions

**Table 2:** Best hospitals by number of mobile apps.

Hospital	Number of Mobile Apps
The Johns Hopkins Hospital, Johns Hopkins Bayview Medical Center (1)	29
University of Michigan Hospitals—Michigan Medicine	17
Cleveland Clinic, Cleveland Clinic Fairview Hospital, Cleveland Clinic Florida (2)	14
Ronald Reagan UCLA Medical Center	11
Vanderbilt University Medical Center, El Camino Hospital, Massachusetts General Hospital	7

(1) John Hopkins Hospital and Johns Hopkins Bayview Medical Center shared the same apps. (2) Cleveland Clinic, Cleveland Clinic Fairview Hospital, and Cleveland Clinic Florida shared the same apps.

**Table 3:** Hospitals and criteria.

Number of Criteria	Number of Hospitals
9	4
8	4
7	50
6	19
5	16
4	1
3	1
2	0
1	0
0	5

**Table 4:** Best apps by number of criteria.

Mobile Apps and Hospitals	Number of Criteria
*UC Health App* (UCHealth University of Colorado Hospital) *My BSW Health App* (Baylor University Medical Center) *M Health Fairview* (University of Minnesota Medical Center) *Christ Hospital Health Network App* (Christ Hospital)	9
*My Mount Sinai App* (The Mount Sinai Hospital) *My UofM Health App* (University of Michigan Hospitals—Michigan Medicine) *My UC Davis Health App* (University of California—Davis Medical Center) *TGH Virtual Health App* (Tampa General Hospital)	8

## Data Availability

The data presented in this study are available on request from the corresponding author.

## Appendix A. List of Best Hospitals in the USA

1. Mayo Clinic—Rochester
2. Cleveland Clinic
3. Massachusetts General Hospital
4. The Johns Hopkins Hospital
5. Stanford Healthcare—Stanford Hospital
6. Ronald Reagan UCLA Medical Center
7. The Mount Sinai Hospital
8. University of Michigan Hospitals—Michigan Medicine
9. Brigham Additionally, Women’s Hospital
10. New York-Presbyterian Hospital-Columbia and Cornell
11. Duke University Hospital
12. Mayo Clinic—Phoenix
13. Cedars-Sinai Medical Center
14. UCLA Medical Center—Santa Monica
15. Northwestern Memorial Hospital
16. Hospital of the University of Pennsylvania—Penn Presbyterian
17. UCSF Medical Center
18. Houston Methodist Hospital
19. Rush University Medical Center
20. Mayo Clinic—Jacksonville
21. NYU Langone Hospitals
22. University of Washington Medical Center
23. UCHealth University of Colorado Hospital
24. Vanderbilt University Medical Center
25. Barnes-Jewish Hospital
26. University of Chicago Medical Center
27. University of California—Davis Medical Center
28. University of Wisconsin Hospitals
29. Scripps Memorial Hospital La Jolla
30. Emory University Hospital
31. Beth Israel Deaconess Medical Center
32. University Hospitals Cleveland Medical Center
33. Yale New Haven Hospital
34. Morristown Medical Center
35. UC San Diego Health—Jacobs Medical Center
36. OHSU Hospital
37. Baylor St. Luke’s Medical Center
38. Mercy Hospital St. Louis
39. Keck Hospital of USC
40. Torrance Memorial Medical Center
41. Cleveland Clinic Fairview Hospital
42. University of Kansas Hospital
43. Hackensack University Medical Center
44. UT Southwestern Medical Center
45. Centra Care—St. Cloud Hospital
46. UAB Hospital
47. Baylor University Medical Center
48. Virginia Mason Medical Center
49. Nebraska Medicine—Nebraska Medical Center
50. Tufts Medical Center
51. University of Virginia Medical Center
52. University of Utah Hospital
53. Memorial Hermann-Texas Medical Center
54. Medical City Dallas Hospital
55. UPMC Presbyterian & Shadyside
56. Loyola University Medical Center
57. Cleveland Clinic—Florida
58. St. Luke’s Regional Medical Center
59. Providence St. Vincent Medical Center
60. Brigham Additionally, Women’s Faulkner Hospital
61. Advocate Good Samaritan Hospital
62. Aurora St. Luke’s Medical Center
63. Miami Valley Hospital
64. St. Luke’s Hospital of Kansas City
65. Inova Fairfax Hospital
66. St. Joseph Mercy Chelsea
67. University of Minnesota Medical Center
68. University of Maryland Medical Center
69. Mayo Clinic—Health System in Eau Claire
70. Christiana Care
71. Tampa General Hospital
72. Jefferson Health—Thomas Jefferson University Hospitals
73. University of Kentucky—Albert B. Chandler Hospital
74. Northwestern Medicine Central DuPage Hospital
75. Ohio State University—Wexner Medical Center
76. Christ Hospital
77. Penn State Health—Milton S. Hershey Medical Center
78. Sharp Memorial Hospital
79. Indiana University Health—North Hospital
80. Johns Hopkins Bayview Medical Center
81. Indiana University Health West Hospital
82. Sanford USD Medical Center
83. VCU Medical Center
84. Inova Alexandria Hospital
85. Penn Medicine Chester County Hospital
86. MUSC Health-University Medical Center
87. Newton-Wellesley Hospital
88. Memorial Care Long Beach Medical Center
89. UnityPoint Health—Meriter
90. Froedtert Hospital and the Medical College of Wisconsin
91. Mission Hospital
92. Reading Hospital
93. Emory Saint Joseph’s Hospital
94. Umass Memorial Medical Center
95. UNC REX Hospital
96. Dartmouth-Hitchcock Medical Center
97. Advocate Lutheran General Hospital
98. El Camino Hospital
99. Hoag Memorial Hospital Presbyterian
100. Baystate Medical Center

Document retrieved on 17 August 2022 from: https://www.newsweek.com/best-hospitals-2021/united-states.

## Appendix B. Hospitals Using the Same Mobile Apps

Some hospitals belong to the same group and therefore use the same mobile apps (general mobile apps, and mobile apps for patients suffering from non-communicable diseases):

- Mayo Clinic Rochester, Mayo Clinic Phoenix, Mayo Clinic Jacksonville, and Mayo Clinic Healthcare System in Eau Claire.
- Cleveland Clinic, Cleveland Clinic Fairview Hospital, and Cleveland Clinic Florida.
- Advocate Good Samaritan Hospital, Aurora St. Luke’s Medical Center, and Advocate Lutheran General Hospital.
- Northwestern Memorial Hospital, and Northwestern Medicine Central DuPage Hospital.
- John Hopkins Medicine, and Johns Hopkins Bayview Medical Center.
- Indiana University Health—North Hospital, and Indiana University Health—West Hospital.
- Inova Fairfax Hospital, and Innova Alexandria Hospital.
- Hospital of the University of Pennsylvania—Penn Presbyterian, Penn Medicine Chester County Hospital, and Penn State Health—Milton S. Hershey Medical Center.
- Brigham Additionally, Women’s Faulkner Hospital, and Brigham And Women’s Hospital.
- Emory Saint Joseph’s Hospital, and Emory University Hospital.

## Appendix C. General Apps from External Companies

Some hospitals use general apps developed by external organizations:

- My Chart (developed by EPIC): UCLA Medical Center—Santa Monica, UCSF Medical Center, Barnes-Jewish Hospital, University of Chicago Medical Center, University of Wisconsin Hospitals, Morristown Medical Center, OHSU Hospital, University of Kansas Hospital, Hackensack University Medical Center, CentraCare—St. Cloud Hospital, Virginia Mason Medical Center, University of Virginia Medical Center, University of Utah Hospital, Loyola University Medical Center, St. Luke’s Regional Medical Center, Providence St. Vincent Medical Center, Miami Valley Hospital, Inova Fairfax Hospital, Jefferson Health—Thomas Jefferson University Hospitals, University of Kentucky—Albert B. Chandler Hospital, MUSC Health-University Medical Center, Memorial Care Long Beach Medical Center, UnityPoint Health—Meriter, Reading Hospital, Umass Memorial Medical Center, UNC REX Hospital, Dartmouth-Hitchcock Medical Center, El Camino Hospital, and Hoag Memorial Hospital Presbyterian.
- Follow My Health (developed by All Scripts): Baylor St. Luke’s Medical Center and Sharp Memorial Hospital.
